# Mumps Outbreak in a Marshallese Community — Denver Metropolitan Area, Colorado, 2016–2017

**DOI:** 10.15585/mmwr.mm6741a2

**Published:** 2018-10-19

**Authors:** Grace E. Marx, Alexis Burakoff, Meghan Barnes, Donna Hite, Amanda Metz, Karen Miller, Emily Spence Davizon, Jennifer Chase, Carol McDonald, Maggie McClean, Lisa Miller, Bernadette A. Albanese

**Affiliations:** ^1^Epidemic Intelligence Service, CDC; ^2^Tri-County Health Department, Greenwood Village, Colorado; ^3^Colorado Department of Public Health and Environment; ^4^Denver Public Health, Colorado.

In January 2017, the Colorado Department of Public Health and Environment (CDPHE) identified four epidemiologically linked cases of mumps among persons from a Marshallese community who were members of the same church in the Denver metropolitan area. During 2016–2017, sizable outbreaks of mumps reported in Arkansas, Hawaii, and Washington also affected the Marshallese population (*1*). CDPHE, the Tri-County Health Department (TCHD), and Denver Public Health collaborated to conduct an outbreak investigation during January–March 2017 using active and passive surveillance that identified 17 confirmed and 30 probable cases. Public health actions included conducting measles-mumps-rubella (MMR) vaccination clinics at local Marshallese churches; these resulted in the vaccination of 126 persons with ≥1 doses of MMR vaccine. Implementation of active surveillance and support from local Marshallese church leaders in promoting vaccination programs likely contributed to interruption of the outbreak.

## Investigation and Results

On January 19, 2017, CDPHE identified a cluster of four mumps cases through routine surveillance in the Denver metropolitan area; the cluster was epidemiologically linked to one local Marshallese church (church A). Initial patient interviews indicated that additional church members had recent symptoms of facial swelling suggestive of mumps. During January 20–22, TCHD staff members met with church A leaders to initiate rapid case ascertainment through active surveillance, and leaders agreed to provide a list of church A member households. Local and state public health staff members attempted to contact each household up to three times by telephone and collected information for each household member, including demographics, reported MMR vaccination history, occurrence and timing of mumps symptoms, travel history, household visitors, and church attendance since November 1, 2016. Cases also were identified through passive surveillance, either from laboratory reports of positive mumps test results in the Colorado Electronic Disease Reporting System or from health care provider reports. A Health Alert Network broadcast was issued to health care providers, and targeted outreach to local hospitals encouraged mumps testing and reporting.

Case definitions were derived from the 2012 Council of State and Territorial Epidemiologists case classification (*2*). A probable outbreak-associated mumps case was defined as the occurrence of mumps-compatible symptoms on or after November 1, 2016, and an epidemiologic link to the Marshallese community in the Denver metropolitan area. A confirmed case was defined as identification of mumps virus by reverse transcription–polymerase chain reaction (RT-PCR) or culture in a person with a probable case. Clinical samples from confirmed cases were sent to CDC for genotyping.

From the contact list of 21 member names provided by church A, 17 members were located, representing 15 unique households (defined as all persons residing at a single address) comprising 117 persons. Interviews were conducted with each head of household on behalf of all household occupants. Information was collected for 76 (65%) household occupants. Median reported household size was six persons (range = 4–18 persons). At least one person from every household included in the interviews attended church A. Among the 76 persons for whom information was collected, 22 (29%) reported attending at least one other Marshallese church gathering since November 1, 2016, in addition to attending church A. Three households reported visitors from Arkansas, the site of a large concurrent mumps outbreak in the Marshallese population (*1*), since November 1, 2016. One visitor from Arkansas reportedly had a “swollen jaw” at the time of the visit in late November 2016.

**FIGURE 1:**
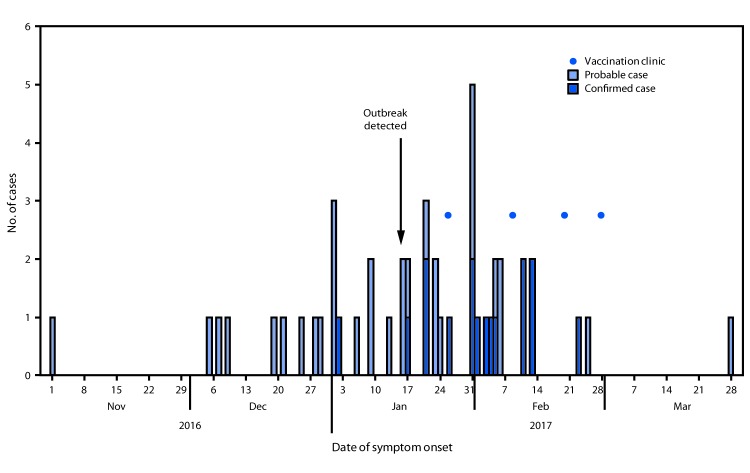
Probable and confirmed cases of mumps (N = 47), by date of symptom onset and measles-mumps-rubella vaccination outbreak response clinics ─ Colorado, 2016–2017

**FIGURE 2:**
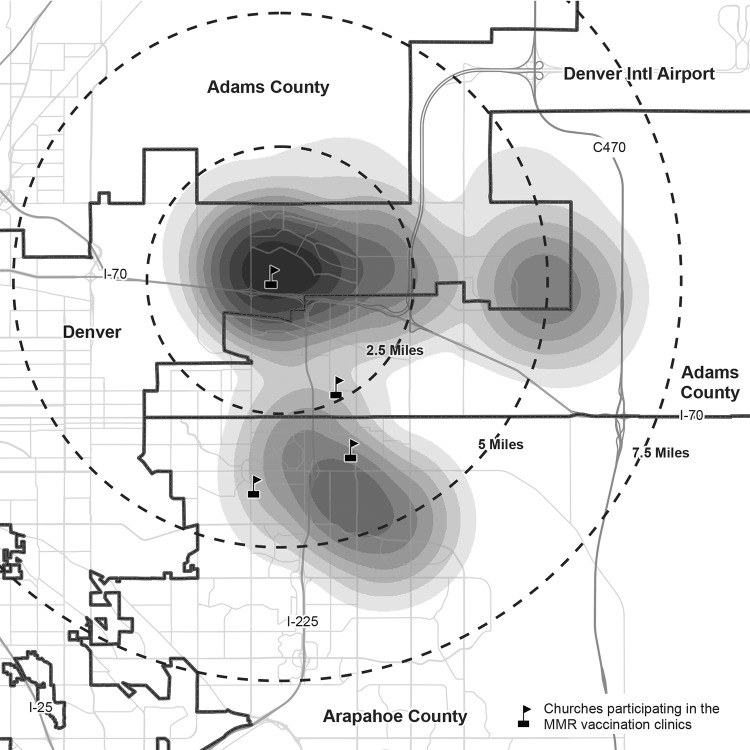
Geographic density* of outbreak mumps cases (n = 46),^†^ by geocoded residential address^§^ and location of measles-mumps-rubella vaccination clinics held during the public health response — Colorado, 2016–2017 * Gradient indicates the relative number of cases; darkest shading indicates highest density. ^†^ One outbreak case occurred in a patient who was geographically isolated from all other cases and was suppressed from the calculation of the density surface to protect patient privacy. ^§^ Density surface is calculated by a kernel density function using the geocoded residential addresses of confirmed mumps cases.

In total, 47 outbreak cases (17 confirmed and 30 probable) were identified, representing two counties in the Denver metropolitan area. Illness onset dates ranged from November 1, 2016, to March 28, 2017 (Figure 1). Among persons with mumps, 24 (51%) were male; median age was 20 years (range = 4 months–44 years; interquartile range = 12–27 years). Forty-six cases (98%) occurred in Marshallese persons. All persons with mumps experienced parotitis; 22 (47%) reported bilateral swelling. Other symptoms included jaw pain (74%), malaise (62%), fever (57%), and submandibular swelling (47%). One pregnant woman, aged 20 years, was hospitalized; no deaths or serious mumps complications (e.g., orchitis, meningitis, or deafness) were reported. Patients were identified from 21 unique households; 15 (71%) households had two or more patients (range two–four). Cases were also tightly clustered geographically; 46 of 47 (98%) patients resided within a 7.5-mile radius (Figure 2). All 17 patients with confirmed cases tested positive for mumps virus by RT-PCR. Samples from 12 patients with confirmed cases were submitted to CDC for genotyping, and all were mumps virus genotype G, the most common genotype currently circulating in the United States (*3*).

Determining accurate MMR vaccination status in this population was challenging. None of the mumps patients was able to provide personal vaccination records, nor were records from outside Colorado available. Thirty-four (72%) of the 47 patients had no confirmed doses of MMR vaccine recorded in the Colorado Immunization Information System, which collects information only on vaccinations administered in Colorado (*4*). One case occurred in an infant aged <12 months who was too young for routine MMR vaccination.

## Public Health Response

After the first cluster of cases was identified, TCHD staff members met with Marshallese community leaders from church A to disseminate information about mumps illness and explore the possibility of hosting MMR vaccination clinics to prevent further transmission. In this Marshallese community, church pastors and their wives served as important spokespersons. After multiple in-person visits with church leaders, TCHD staff members organized four vaccination clinics at four different Marshallese church locations (including church A), during the 5 weeks after the outbreak was recognized; the first clinic was held at Church A, 9 days after identification of the outbreak (Figure 1). The goal of the immunization clinics was to offer all eligible persons from the Marshallese community up to 2 MMR doses; vaccine eligibility was determined by age and documented vaccination history, according to Advisory Committee on Immunization Practices (ACIP) recommendations at the time of the response (*4*). For children aged 12 months—4 years, an accelerated second MMR dose was given if the child previously received only 1 documented dose at least 28 days earlier.

In total, 164 MMR vaccine doses were administered to 126 church attendees; 38 (30%) persons received 2 doses, administered at least 28 days apart per ACIP guidelines, during clinics. Median age of church attendees who were vaccinated at the clinics was 20 years (range = 1–55 years).

In addition to vaccination clinics, local public health agencies disseminated culturally sensitive messages regarding mumps disease and prevention through Marshallese church leaders, radio, and social media; and CDPHE issued two press releases to alert the public about mumps. Local public health staff members contacted affected school districts in the Denver metropolitan area and provided a letter for parents urging them to have children without 2 documented doses of MMR vaccine receive catch-up vaccination, according to ACIP guidelines. The outbreak was declared over on May 17, 2017, 50 days (two incubation periods) after the last reported case.

## Discussion

This mumps outbreak occurred in a Denver metropolitan area Marshallese community characterized by a strong cultural and social network with frequent community gatherings. Regular socializing and large households likely contributed to mumps transmission throughout Marshallese households and churches. Although mumps importation from another state could not be confirmed, it is likely that mumps was introduced into this community from out-of-state Marshallese persons, given frequent travel to and from areas with concurrent mumps outbreaks in other Marshallese communities. The outbreak did not spread widely outside the Marshallese community in the Denver metropolitan area. Only one case in a non-Marshallese person was identified at a school attended by multiple Marshallese patients.

Public health staff members used active surveillance after identification of the initial mumps cluster to identify additional cases and to assess potential contributing factors for transmission, including household size and travel history. Early and close communication between TCHD staff members and church leaders helped to inform the affected population about the risks for mumps. As a result, a substantial number of Marshallese persons in the Denver area received MMR vaccine. The willingness of the community to receive MMR vaccine with support from church leaders highlights the importance of these community partnerships.

The vaccination clinics held in response to this outbreak focused on vaccinating persons who did not have 2 previously documented doses of MMR. At the time of this outbreak, ACIP did not recommend a third MMR dose in an outbreak setting for previous 2-dose recipients (*4*,*5*). In addition, the effectiveness of a third dose for an outbreak in a population with undocumented vaccination history, such as this one, was unclear; use of a third dose had been described primarily among populations with high documented 2-dose MMR coverage, such as college students (*6*–*8*). However, since this outbreak, ACIP guidelines were updated to recommend a third dose of mumps virus–containing vaccine for persons previously vaccinated with 2 doses and who are identified by public health authorities as being part of a group or population at increased risk for acquiring mumps because of an outbreak (*5*).

The findings in this report are subject to at least three limitations. First, vaccination status was determined primarily through Colorado’s immunization registry, which likely did not record all previously received vaccines in this highly mobile community. Persons previously vaccinated in the Republic of the Marshall Islands or another state might have been misclassified as being unvaccinated, which highlights the need for interoperable state registries. Second, language and cultural barriers might have led to errors in collecting information, especially during telephone interviews, despite use of interpreters and translated materials. Finally, uncertainty regarding household living arrangements made accurate identification of household members a challenge and might have resulted in an underestimation of household sizes and response rate.

Response to this mumps outbreak in a Colorado Marshallese community was facilitated by building relationships with church leaders, leading to early active surveillance, public education, and MMR vaccination clinics. These interventions might have contributed to the rapid interruption of transmission and limited spread of mumps to other local communities.

**Summary**

**What is already known about this topic?**

Mumps outbreaks typically occur among persons in close contact, such as in schools and athletic teams. Measles-mumps-rubella (MMR) vaccine can prevent mumps.

**What is added by this report?**

An outbreak of 47 mumps cases occurred in the Denver metropolitan area, mostly among members of a Marshallese community. Public health response included early active surveillance, public education, and prompt implementation of MMR vaccination clinics.

**What are the implications for public health practice?**

All eligible children should receive MMR vaccine at age 12–15 months and 4–6 years. During a mumps outbreak, eligible persons should receive prompt MMR vaccinations according to Advisory Committee on Immunization Practices guidelines, including use of a third dose when appropriate.
